# Defective pollen wall contributes to male sterility in the male sterile line 1355A of cotton

**DOI:** 10.1038/srep09608

**Published:** 2015-06-05

**Authors:** Yuanlong Wu, Ling Min, Zancheng Wu, Li Yang, Longfu Zhu, Xiyan Yang, Daojun Yuan, Xiaoping Guo, Xianlong Zhang

**Affiliations:** 1 National Key Laboratory of Crop Genetic Improvement, Huazhong Agricultural University, Wuhan, Hubei 430070, China

## Abstract

To understand the mechanisms of male sterility in cotton (*Gossypium* spp.), combined histological, biochemical and transcription analysis using RNA-Seq was carried out in the anther of the single-gene recessive genic male sterility system of male sterile line 1355A and male fertile line 1355B, which are near-isogenic lines (NILs) differing only in the fertility trait. A total of 2,446 differentially expressed genes were identified between the anthers of 1355AB lines, at three different stages of development. Cluster analysis and functional assignment of differentially expressed genes revealed differences in transcription associated with pollen wall and anther development, including the metabolism of fatty acids, glucose, pectin and cellulose. Histological and biochemical analysis revealed that a major cellular defect in the 1355A was a thicker nexine, consistent with the RNA-seq data, and further gene expression studies implicated differences in fatty acids synthesis and metabolism. This study provides insight into the phenotypic characteristics and gene regulatory network of the genic male sterile line 1355A in upland cotton.

Cotton (*Gossypium* spp.) is one of the most important economic crops due to the value of its fibre. Heterosis is one strategy to improve cotton fibre productivity and quality. Male sterility, as an effective and economical pollination control system, is used worldwide for the exploitation of cotton heterosis. There have been identified 17 types of genic male sterile (GMS) lines in cotton^1^, among which 1355A belongs to the single-gene recessive GMS system. In the 1355A male sterile plant, the exine spine is not produced at the uninucleate stage, and the thickness of the pollen wall increases^2^. However, the pollen wall fine structure defect was still unknown. Physiological and biochemical analysis indicates that starch levels are lower in male sterile plants throughout the entire period of pollen development, and the soluble sugar level in young stamen of sterile plants are lower than in fertile plants, but the opposite is the case in mature stamens^1^. Recently RNA-seq was used to investigate the ‘Dong A’ male sterile mutant, showing that several key branch-point genes involved in hormone signaling, carbon and energy metabolism and pollen wall development were differentially expressed in the ‘Dong A’ male sterile mutant anther^3^. However, relatively little has been reported on the molecular genetic mechanism of 1355A male sterility.

In flowering plants, the haploid male sperm cells are protected in the pollen grains by the complex cell walls that are divided into two layers: the exine and the intine. The exine is a multilayered structure that is divided into sexine and nexine, and is primarily composed of sporopollenin, composed of fatty acids and phenolics^4^,^5^,^6^. The intine is structurally simpler and is composed of cellulose, hemicellulose, pectin polymers, hydrolytic enzymes and hydrophobic proteins^7^,^8^,^9^.

Pollen wall development begins in the newly formed microspores within the tetrad that are covered by the callose wall, which compartmentalises individual microspores when the primexine is laid down^10^. The primexine is the site of self-assembly of the glycocalyx and sporopollenin receptors^11^. The primexine has been described as a sporopollenin receptor because of the organised manner in which it accumulates sporopollenin at specific places^12^. During the tetrad stage, an undulating surface structure is formed in the plasma membrane of the microspore, which might be associated with normal primexine formation^13^. At the end of the tetrad stage, the microspores are released from the callose wall, and the exine exhibits a considerable increase in thickness because of the increased deposition and polymerisation of tapetally derived sporopollenin precursors^9^. In addition, the spines and other features of pollen ornamentation, which can be observed in some plants such as cotton, also appear during this stage. The mature exine structure is anatomically complete by the bicellular pollen stage. When the tapetum degrades, the remnants deposit as tryphine to fill the exine cavities, and the mature pollen wall is formed.

Recent molecular genetic studies have increased our understanding of pollen wall development. For example in *Arabidopsis*, *CALLOSE SYNTHETASE5* (*CALS5*) is involved in callose synthesis^14^; *NO EXINE FORMATION1* (*NEF1*), *DEFECTIVE IN EXINE FORMATION1* (*DEX1*) and *NO PRIMEXINE AND PLASMA MEMBRANE UNDULATION* (*NPU*) play critical roles in primexine formation^15^,^16^,^17^; and *MALE STERILITY1* (*MS1*), *DIHYDROFLAVONOL 4-REDUCTASE-LIKE1/TETRAKETIDE–PYRONE REDUCTASE1* (*DRL1/TKPR1*), *ECERIFERUM1* (*CER1*), *MALE STERILITY2* (*MS2*), *FACELESS POLLEN1* (*FLP1*), *RUPTURED POLLEN GRAIN1* (*RPG1*), *ACYL-COA SYNTHETASE5* (*ACOS5*), *CYTOCHROME P450 703A2* (*CYP703A2*) and *CYTOCHROME P450 703B1* (*CYP704B1*), *TETRAKETIDE–PYRONE REDUCTASE2* (*TKPR2*), *LAP6/POLYKETIDE SYNTHASE A* (*PKSA*), and *LAP5/POLYKETIDE SYNTHASE B* (*PKSB*) are required for exine formation^18^,^19^,^20^,^21^,^22^,^23^,^24^,^25^,^26^,^27^,^28^.

Here, we report a transcriptome profiling analysis of anther development in the upland cotton male sterile line 1355A compared to that of the male fertile line 1355B at three different stages, using RNA-Seq. By identification of differentially expressed genes (DEGs), we identified pathways altered in 1355A male sterility. Anatomical studies show that the thicker nexine in 1355A contributes to male sterility and are consistent with pathways implicated by the RNA-seq data analysis, and suggest abnormal sporopollenin synthesis and deposition might lead to male sterility in 1355A.

## Results

### Phenotypic characteristics of the 1355A line

**Figure 1:**
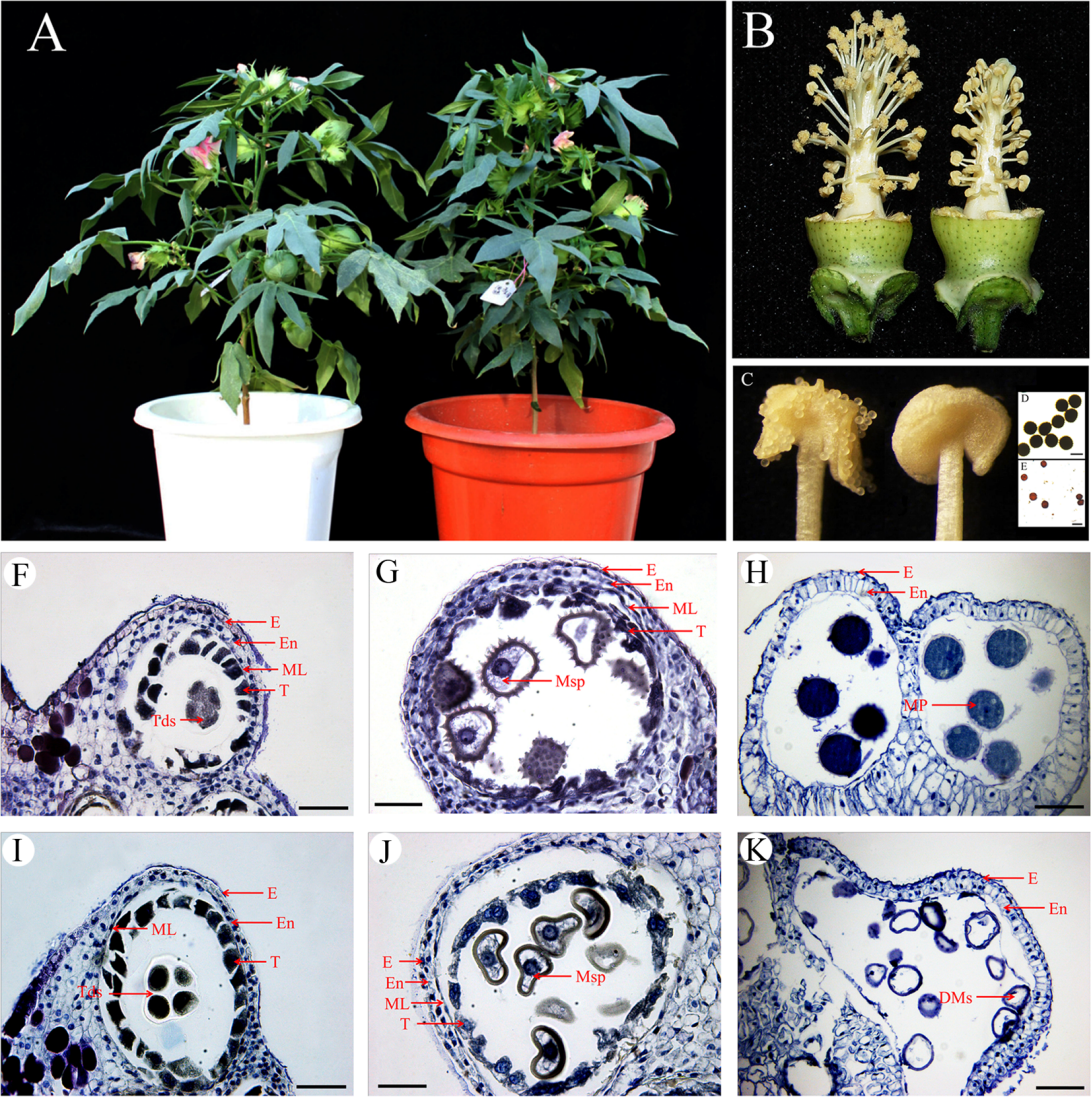
Phenotypic comparison between the 1355B and the 1355A lines. (A) A 1355B plant (left) and a 1355A plant (right) at full-bloom stage. (B) A 1355B flower (left) and a 1355A flower (right), with petals removed. (C) In a 1355B anther (left), dehiscence is normal; while in a 1355A anther (right), dehiscence is abnormal. (D) 1355B pollen grains stained with 1% I_2_-KI solution at stage 12 showing mature pollen grains that are dyed black. (E) 1355A pollen grains stained with 1% I_2_-KI solution at stage 12 showing mature pollen grains that are not dyed black. (F–K) Locules from the anther section of the 1355B (F–H) and 1355A (I–K) plants during stage 7, stage 8 and stage 12. (F) and (I) stage 7. (G) and (J) stage8. (H) and (K) stage 12. There are no differences between the 1355B (F) and 1355A (I) plants at stage 7. Compared to those of the 1355B plant (G), the spines could not be identified on the surface of pollen in the 1355A plant (J). The microspore cytoplasm was stained deeply in the 1355B plants (H), but the microspores aborted in 1355A plants (K). E, epidermis; En, endothecium; ML, middle layer; T, tapetal layer; Mp, mature pollen; DMs, degenerated microspores; Msp, microspores and Tds, tetrads. Bars, 100 μm in (D) and (E); Bars, 50 μm in (F) to (K). Acknowledge the authors Yuanlong Wu and Li Yang for photographing (A), Yuanlong Wu for photographing (B), (D) and (E), and Zancheng Wu for photographing (C) and (F) to (K).

Morphological comparison of lines 1355A and 1355B revealed that the vegetative and floral development appears normal in 1355A plants (Fig. 1A). However, stamen filaments were shorter in 1355A (Fig. 1B) and anther dehiscence was abnormal (Fig. 1C) with pollen grains failing to show staining by I_2_-KI (Fig. 1D and E).

To identify the relationship between bud length (from the nectary to the top of the bud) and anther development stages, cross sections were performed on the 1355B anther. Fourteen stages of anther development were identified based on the distinctive cellular events of *Arabidopsis thaliana* and *G. hirsutum* (Coker 315) anther development stages^29^,^30^ (Supplementary Table S1 and Supplementary Fig. S1).

To investigate the cellular defects of the 1355A male sterile plants during pollen development, cross sections were compared to those of the 1355B anther at stage 7, stage 8 and stage 12, referred to our previous observation that the first detectable sign of male sterility occurs at stage 8 in the 1355A. And in order to know what changed in mature pollen, we selected stage 12 when the pollen is mature. No detectable differences were observed between the 1355B and the 1355A anthers prior to stage 7 (Fig. 1F and I), while significant differences in anther development were observed after the tetrad stage (stage 8). When the microspores of 1355B plants were released from the callose walls, the microspores were normal, and the exine with spines was formed (Fig. 1G). In contrast, the 1355A microspores were shrunken in appearance, and the exine lacked spines (Fig. 1J). On the day of anthesis (stage 12), the cytoplasm of mature pollen of 1355B anthers stained strongly with toluidine blue (Fig. 1H), while 1355A pollen cytoplasm was empty, and only residual defective pollen walls were present in the anther locules (Fig. 1K), which means the cytoplasm is degraded.

### Identification of the genes that were differentially expressed between lines 1355A and 1355B

Previous studies and our phenotypic observations indicated that the first detectable sign of male sterility occurs during the early uninucleate microspore stage (stage 8) in the 1355A plants. Therefore, anthers were collected at the tetrad stage (stage 7), the uninucleate microspore stage (stage 8) and the mature pollen stage (stage 12) with a view to identifying differentially expressed genes (DEGs) between 1355B and 1355A, using RNA-Seq technology.

The results of the classification of raw reads and randomness assessment indicate that high-quality libraries were generated (Supplementary Fig. S2A and B). The six libraries include a total of 44,391,107 clean reads (Table 1), and each read was approximately 50 bp in length. A sequencing saturation analysis indicated that up to 7.3 million clean reads for each sample were created, which is sufficient for the quantitative analysis of gene expression (Supplementary Fig. S2C). By mapping the read sequences to a reference database (cotton unigenes from NCBI) containing 20,671 unigene sequences, we obtained a total of 16,949,140 (38.18%) mapped reads, but there were only 15,473,443 (34.86%) unique match reads and the number of unique match reads ranged from 2,399,000 to 2,885,941 in six libraries. A total of 27,441,967 (61.82%) clean reads were not mapped to the cotton unigenes assembly (Table 1).

**Figure 2:**
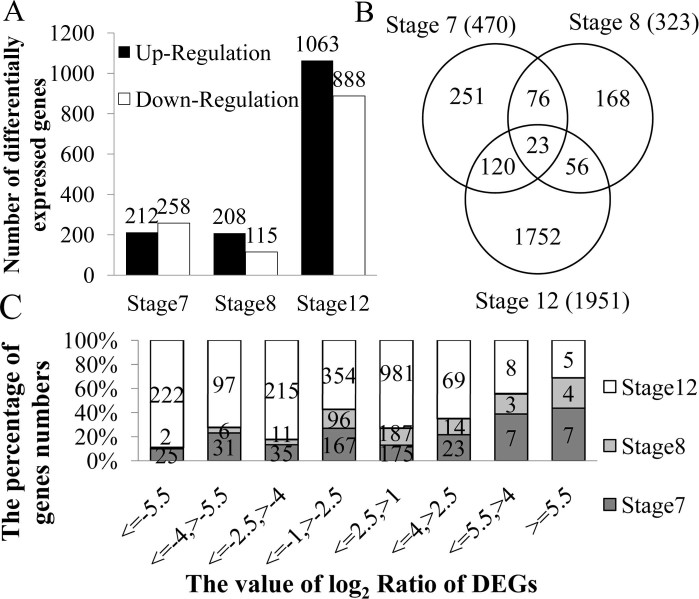
The statistical analysis of differentially expressed genes in the 1355A plants compared to those of the 1355B plants. (A) Number of differentially expressed genes that were up- or down-regulated during analyzed stages. (B) Showing the relationship of differentially expressed genes in three pollen development stages using a Venn diagram. The overlapping regions indicate the number of DEGs that are present in more than one stage, and the central region corresponds to the expressed genes that are present in all three of the stages. (C) The distribution of the value log_2_ Ratio of the DEGs in the 1355A plants compared to the 1355B plants.

The expression levels of genes were determined by calculating the number of unique match reads for each gene and then normalising this number to Reads Per Kb per Million reads (RPKM), which associates the read numbers with the gene expression levels. A total of 20,113 genes were detected during the three stages of anther development in the 1355AB lines (Supplementary Table S2-1). Among these genes, 2,446 differentially expressed genes were filtered with FDR ≤ 0.001 and absolute value of log_2_ Ratio ≥ 1 (Supplementary Table S2-2). The number of DEGs that were up- or down- regulated during different pollen development stages demonstrates that the least number of the DEGs occurs at stage 8 rather than at stage 7, and at stage 7, more down-regulated DEGs than up-regulated DEGs were identified. At stage 8 and stage 12, more up-regulated DEGs than down-regulated DEGs were identified (Fig. 2A). This may indicate that the reversed up- and down- regulated DEG ratios at stage 7 and stage 8 contains critical information linked to male sterility.

The number of DEGs during analysed stages was 470, 323 and 1,951 (Fig. 2B). Among these genes, only 23 were present in all three of the stages. However, more than half of the DEGs exhibited specific expression in the three stages of anther development (Fig. 2B). By analysing the distribution of the DEGs log_2_ Ratio, we found that many DEGs have an absolute value of log_2_ Ratio range from 1 to 2.5 during stages 7 and 8. During the stage 12, the expression of the down-regulated DEGs was distributed widely (Fig. 2C). This result indicates that the male sterility gene affects little transcriptional changes in 1355A anther.

Using BLASTx against the non-redundant protein sequence (nr) database in GenBank, 2,446 DEGs were annotated. Among these, 1,793 genes (73.30% of the differentially expressed sequences) had an above-the-cut-off BLAST result; 436 genes (17.83%) belonged to the functional categories ‘unknown proteins’ or ‘predicted proteins’; 217 genes (8.87%) had ‘no hits’ results (Supplementary Table S2-2). However, 2,138 genes (87.41%) had an orthologue in Arabidopsis (Supplementary Table S3).

To evaluate the potential functions of the DEGs, we added gene ontology (GO) terms to DEGs at stages 7 and 8 (Supplementary Fig. S3A and B). In terms of annotated biological processes, the difference between these genes was ‘cellular process’ (16% at stage 7 and 13% at stage 8) at level 2. This biological process was further studied at level 3. The results indicate that the ‘cellular metabolic process’ is significantly different to ‘cellular process’ (Supplementary Fig. S4A and B). Interestingly, ‘cellular homeostasis’ is particularly highlighted during stage 8 (Supplementary Fig. S4B), suggesting a role for the male sterility defect in anther metabolic homeostasis at stage 8.

### Genes those are associated with male sterility in 1355A plant anthers

The bioinformatics analysis indicates that several subsets of genes, including those associated with metabolic events and cell wall-related genes, are differentially expressed during 1355B and 1355A anther development associated with male sterility.

### Metabolic events

Basic metabolic events play an essential role in anther development^31^. Many metabolic genes were identified amongst the DEGs, particularly fatty acid biosynthesis-related genes, fatty acid metabolism-related genes and glucose- and phenylpropanoid pathway- related genes.

#### Fatty acids

Using the KEGG results and other annotations, several genes participating in fatty acid biosynthesis and fatty acid metabolism were identified by RNA-Seq analysis. Seven fatty acid biosynthesis genes, namely fatty acyl-ACP thioesterases B (ES849077), NAD(P)-binding Rossmann-fold superfamily protein (ES818872), acetyl-CoA carboxylase 1 (ES840243 and ES842786) and acetyl-CoA carboxylase (ES805503, ES796810 and DW482723), were down-regulated at stage 7 in the 1355A anthers (Supplementary Table S4-1). One gene encoding an NAD(P)-binding Rossmann-fold superfamily protein (ES806303) was up-regulated at stage 7 and stage 8 but down-regulated at stage 12. Interestingly, the expression patterns of fatty acid metabolism genes were different from those of fatty acid biosynthesis. Five fatty acid metabolism genes were up-regulated in the 1355A at stage 7, these encoded acyl-CoA oxidase 3 (EX164385), long-chain acyl-CoA synthetase 6 (ES813531), aldehyde dehydrogenase 3I1 (DW480953), GroES-like zinc-binding alcohol dehydrogenase family protein (ES797791) and acetoacetyl-CoA thiolase 2 (ES834402) (Supplementary Table S4-1).

#### Glucose

Subsets of genes participating in multiple branches of the energy metabolism pathway, including starch and sucrose metabolism, glycolysis/gluconeogenesis and pyruvate metabolism, were identified. The expression patterns of the starch and sucrose metabolism genes were up-regulated during analyzed stages in the 1355A line, although four genes were down-regulated, including the gene of UDP-D-glucuronate 4-epimerase 3 (GQ292790; Supplementary Table S4-2). In contrast, the expression patterns of glycolysis/gluconeogenesis genes were mostly down-regulated at stage 7 (Supplementary Table S4-2). Genes are associated with pyruvate metabolism were up-regulated during analyzed stages in the 1355A, similar to the genes involved in starch and sucrose metabolism (Supplementary Table S4-2).

### Cell wall component: pectin and cellulose

Genes related to cell wall components were identified. Down-regulated in the 1355A were three cellulose synthase genes (ES811740, ES815864 and ES805098), seven pectin lyase-like superfamily protein genes (EX164190, DW484863, EX166022, U09717, EX165880, ES831677 and ES804623), two plant invertase/pectin methylesterase inhibitor superfamily genes (EX164972 and EX165781) and one pectin methylesterase gene (DT560826; Supplementary Table S5-1). These genes might contribute to the change of intine formation in 1355A.

### Comparison of DEGs data with qRT-PCR

To confirm the expression profiles in the 1355B and 1355A anthers obtained by RNA-seq, qRT-PCR was performed on 17 genes, including fatty acid biosynthesis genes (Supplementary Fig. S5A), fatty acid metabolism genes (Supplementary Fig. S5B), pectin-related genes (Supplementary Fig. S5C) and other metabolic-related genes (Supplementary Fig. S5D), during the three anther development stages.

Pearson's correlation coefficient was calculated by SPSS to assess the correlation between different platforms. Overall, the 29 DEGs of the qPCR measurements were moderately correlated with the RNA-Seq results (Supplementary Fig. S6. A, R = 0.854; correlation is significant at the 0.01 level). The correlations during analyzed stages were 0.578, 0.909 and 0.914 (Supplementary Fig. S6B to D). These results indicate that the RNA-seq method is an accurate and reliable way to identify genes that are differentially expressed during cotton anther development.

### The defective pollen wall in the 1355A male sterile line

**Figure 3:**
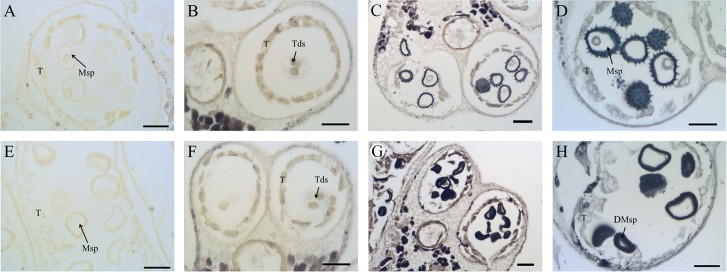
Sudan Black B staining of anthers of the 1355B and1355A plants. The black staining signals indicate the lipid accumulation. (A) and (E) The non-stained anthers of the 1355B (A) and 1355A (E) plants at stage 8 are the negative signals. (B) and (F) The stained anthers of the 1355B (B) and 1355A (F) plants at stage 7. (C) and (G) The stained anthers of the 1355B (C) and 1355A (G) plants at stage 8. (D) and (H) The stained anthers of the 1355B (D) and 1355A (H) plants at stage 9. T, tapetal layer; Tds, tetrads; Msp, microspores; DMsp, degenerated microspores; Bars = 50 μm.

The RNA-seq results suggest that lipid metabolism in the 1355A anther might be abnormal. To confirm this, the lipid stain Sudan black B was used to stain the anther cross sections of the 1355AB plants with non-stained anther cross sections at stage 9 as negative controls (Fig. 3A and E). At stage 7, weak positive staining was detected in the tetrads of both the 1355B and 1355A anthers (Fig. 3B and F). Compared with 1355B (Fig. 3C), the staining sharply accumulated in the pollen wall of 1355A microspores at stage 8 (Fig. 3G). At stage 9, the staining was strong in both the 1355B and 1355A microspores (Fig. 3D and H). This result suggests that abnormal lipid metabolism be associated with abnormal pollen wall development at stage 8 in the 1355A anther. SEM analysis also demonstrates that 1355A anthers exhibit defective pollen walls, whereby the mature pollen wall lacks spines, consistent with the observations from the cross sections (Supplementary Fig. S7).

**Figure 4:**
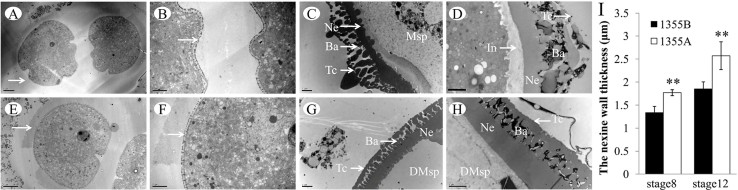
TEMS of pollen walls from the 1355B and 1355A plants. (A) and (E) Anthers of the 1355B (A) and 1355A (E) plants at stage 7 showing tetrads. The white arrows indicate the callose wall of the tetrads. (B) and (F) The higher magnification of (A) and (E) respectively. The white arrows indicate primexine and probacula formation. (C) and (G) Pollen wall of the 1355B (C) and 1355A (G) plants at stage 8 exhibiting early uninuclear microspores. (D) and (H) Pollen wall of the 1355B (D) and 1355A (H) plants at stage 12 exhibiting mature microspores. (I) The nexine wall thickness at stages 8 and 12. Msp, microspores; DMsp, degenerated microspores; In, intine; Ne, nexine; Ba, bacula; Te, tectum. The error bars represent SD (student's t test, ** p < 0.01, p = 5.34E-07 at stage 8, p = 1.39E-05 at stage 12). Bars, 5 μm in (A) and (E); Bars, 2 μm in (B) to (D), and (F) to (H).

To further study the defects in the 1355A pollen wall formation, the fine structure of the pollen wall in the 1355AB plants was observed by TEM. At tetrad stage, characteristic callose walls (Fig. 4A and E) and the formation of primexine (Fig. 4B and F) were detected in both 1355B and 1355A microspores. As development proceeded, the basic exine structure, with an undulating sexine formed at the young uninucleate pollen stage, was seen in 1355B microspores (Fig. 4C). However, a thickened nexine and smooth sexine were observed in the 1355A microspores at this stage (Fig. 4G). There was obvious intine in the mature 1355B pollen (Fig. 4D); however, the 1355A pollen intine was not formed (Fig. 4H). Direct measurements showed that the pollen nexine of 1355A is thicker than that of 1355B (Fig. 4I).

### Homologous genes of pollen wall development

**Figure 5:**
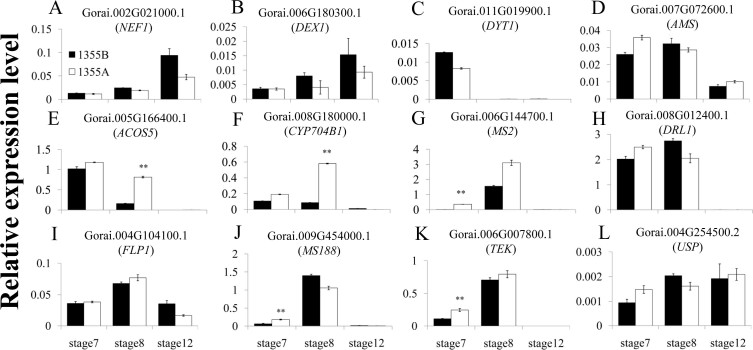
The expression pattern of homologous genes in pollen development. (A) and (B) The primexine formation genes. (C) and (D) The tapetum development genes. (E) to (I) The sporopollenin synthesis-related genes. (J) The sexine formation genes. (K) The nexine formation gene. (L) The intine synthesis gene. The white and black columns refer to the 1355A male sterile line and 1355B male fertile line, respectively. The error bars represent SD (student's t test, ** p < 0.01 and absolute value of log_2_ Ratio ≥ 1, p = 8.06E-07 in (E), p = 8.16E-09 in (F), p = 1.73E-05 in (G), p = 3.34E-04 in (J) and p = 5.64E-04 in (K)). Three biological replicates were performed.

To further explore the molecular mechanism of male sterility in the 1355A mutant, expression of homologous genes from *Arabidopsis thaliana*, associated with pollen wall development, was analysed by qRT-PCR in both cotton lines. Expression of the primexine genes *NEF1* and *DEX1* was not significantly differentially expressed between the 1355A and 1355B anthers (Fig. 5A and B). The tapetum development genes *DYT1* and *AMS* also were not significantly differentially expressed between the 1355A and 1355B anthers (Fig. 5C and D). However, the sporopollenin synthesis-related genes *ACOS5* and *CYP704B1* were differently expressed at stage 8 between 1355B and 1355A (Fig. 5E and F), and the *MS2* gene was differently expressed at stage 7 between 1355B and 1355A (Fig. 5G). Interestingly, the substrates of *CYP704B1*, *ACOS5* and *MS2,* fatty acids and the fatty acids derivatives are also constituents of the pollen wall. The sporopollenin synthesis-related genes *DRL1* and *FLP1* did not exhibit significant differential expression between 1355B and 1355A (Fig. 5H and I). The *MS188* and *TEK* for sexine and nexine formation, respectively, were differently expressed at stage 7 between 1355B and 1355A (Fig. 5J and K). However, the UDP-sugar pyrophosphorylase (*USP*) gene for intine synthesis did not exhibit significant differential expression between 1355B and 1355A (Fig. 5L).

## Discussion

Through comparing the definition of anther development stages in *Arabidopsis thaliana* and *G. hirsutum* of Coker 315 line and 1355B line (Supplementary Table S1), we conclude that cotton anther development is similar to that of *Arabidopsis thaliana*. One important difference between anther development in cotton and *Arabidopsis thaliana* is that the tapetal cell degradation commences earlier in cotton than in *Arabidopsis thaliana* (Supplementary Table S1), the other is the structure of the pollen wall. *Arabidopsis thaliana* pollen has a reticulate structure^9^, while the pollen wall of cotton is more complex. The mature pollen is generally spiny in the exine^32^, similar to the family Compositae^33^. These results, which are similar by identifying the anther development stages in G. *hirsutum* (Coker 315 and 1355B) in different indicative sizes, provide a lot of information for the cotton anther development research.

In this study, an RNA-seq bioinformatics-guided histological analysis reveals that the lack of the spines, as pollen wall ornamentation, is a hallmark of the pollen wall of the 1355A male sterile line. Further studies suggest that defective metabolic events, particularly fatty acid, affect pollen wall formation in the mutant.

The pollen wall functions as a barrier against unfavourable environment conditions and maintains pollen viability^34^, and the exine, which is divided into sexine and nexine, and the intine are the two principal layers of the pollen wall^35^. Previous studies indicate that the *AMS* gene directly regulated the genes of *MS188* and *TEK* in *Arabidopsis thaliana*, which are the key regulators for the sexine and nexine formation respectively^36^,^37^. In our study, we did not detect significantly different in the cotton homologues of *AMS* expression, while the expression patterns of *MS188* and *TEK* homologues were significantly up-regulated at stage 7 (Fig. 5J and K), in agreement with the histological analysis results that show a lack of ornamentation (spines) in the sexine (Fig. 4G) and thicker nexine (Fig. 4I) in the 1355A pollen wall. For the *MS188* and *TEK* homologues, two transcriptional regulation factors, they may lead to the phenotype delay upon the gene expression change happened. This is the reason why the *MS188* and *TEK* homologues showed significantly different in expression at stage 7, while the phenotypes were present at stage 8. We propose that the up-regulated expression of *MS188* and *TEK* homologues at stage 7 might affect the sexine lacking spines and the thicker nexine formation at stage 8 in 1355A, respectively. These results may indicate that the male sterile gene may act as a repressor for the *MS188* and *TEK*, which is independent of *AMS*.

The main composition of the exine is sporopollenin^9^. Sporopollenin synthesis requires many genes that are precisely regulated. Several studies have demonstrated that the *ACOS5* gene is most highly expressed during anther development at stage 8 in *Arabidopsis thaliana*^21^; the *CYP704B1* gene is mainly expressed in flower buds at stage 9 and 10 (corresponding to the anther development from stage 5 to 9) in *Arabidopsis thaliana*^24^, and the expression of *MS2* is initiated at stage 7^38^. These results are consistent with the expression pattern of cotton homologues in 1355B (Fig. 5E to G). The mutations in *Arabidopsis thaliana* *ACOS5* gene cause an apparent loss of exine pollen wall^21^; Mutations in the *Arabidopsis thaliana* *CYP704B1* gene cause a lack of a normal exine layer and a striped surface^24^, and the mutations in the *Arabidopsis thaliana* *MS2* gene caused a poorly constructed pollen wall^38^. These results indicate that the down-regulation of the *ACOS5*, *CYP704B1* and *MS2* genes leads to decreased sporopollenin biosynthesis. Interestingly, in the 1355A anthers, the expression patterns of *ACOS5* and *CYP704B1* homologues were dramatically up-regulated at stage 8, compared to the 1355B anthers (Fig. 5E and F), and the expression patterns of *MS2* homologue was also significantly up-regulated at stage 7 (Fig. 5G). The expression change of *ACOS5*, *CYP704B1* homologues, which is in agree with the stage of phenotype emerged, was more dramatically than *MS2* homologue, which indicated the *ACOS5*, *CYP704B1* homologues were more important than *MS2* homologue for the phenotype of 1355A. We propose that some sporopollenin synthesis genes that are up-regulated at stage 8 might lead to excessively accumulating sporopollenin and finally to male sterility.

While the *DRL1* gene is involved in pollen wall development^23^ and the *FLP1* gene is involved in sporopollenin biosynthesis during the latter stages of anther development^17^, there were no significant differences (Fig. 5H and I) between these 1355A and 1355B anthers; it seems that the transcriptional regulation of these genes is not relevant to the 1355A male sterility phenotype.

We also found that the intine was lacking in the 1355A pollen (Fig. 4D and H). The intine is composed of hydrolytic enzymes, hydrophobic proteins, cellulose, hemicellulose, and pectic polymers^39^. In addition, the microspore cytoplasm was degraded, and the 1355A pollen only maintained the pollen wall after the later uninucleate pollen stage, which might be partly explained by most protease genes being up-regulated during the late development stage in the 1355A anthers (Supplementary Table S5-2), and cell wall-related genes, such as pectin and cellulose genes, were down-regulated (Supplementary Table S5-1). However, the homologue of UDP-sugar pyrophosphorylase (*USP*), which is important in intine synthesis^40^, was no significant differences (Fig. 5L) between 1355A and 1355B anthers. Previous studies indicated that the nexine acts as a template for the intine formation^37^, in agreement with our results. We propose that laking of intine possibly is not the direct reason for the 1355A male sterility, but the thicker nexine.

The production of a functional pollen wall requires precisely coordinated metabolic events^33^, particularly fatty acid, pectin and glucose metabolism. Due to anther development requiring large amounts of sugar^41^, it has been proposed that sporopollenin, which is the main exine wall component, may contain derivatives of aliphatics, such as fatty acids derivatives and phenolics^5^,^42^,^43^. During pollen development, the pollen wall is rich in lipids^5^,^42^,^44^. Several studies have demonstrated that fatty acids are subjected to hydroxylation by *CYP703B1*^24^. Hydroxylauric acid serves as a substrate for *ACOS5*, which is available for conversion to hydroxylauryl-CoA^21^. The *MS2* gene product converts hydroxylauryl-CoA to fatty alcohols, which are the sporopollenin monomeric constituents^38^. Our RNA-seq results indicate that, in 1355A anthers the *FATB* gene (ES849077) and the *ACC1* gene (ES840243) were down-regulated compared to 1355B anthers at stage 7; one *ENR1* (ES806303) gene was up-regulated at stage 7, while a second *ENR1* gene (ES818872) was down-regulated at stages 7 and 8 but up-regulated at stage 12. These genes are related to fatty acid biosynthesis. Other studies have also demonstrated that the *FATB* fatty acid biosynthesis gene (ES849077) mutant reduces the cytosolic supply of palmitate^45^ and the levels of straight-chain C20-24 components^46^ in *Arabidopsis thaliana*. The *FATB* gene also produces free fatty acids for sporopollenin biosynthesis^47^. The *ACC1* fatty acid biosynthesis gene (ES840243) catalyses the rate-limiting step of fatty acid de novo biosynthesis^48^. The *ENR1* fatty acid biosynthesis gene is a subunit of the fatty acid synthase complex^49^. In contrast, in 1355A anthers, the *ACX3* fatty acid metabolism gene (EX164385) was up-regulated at stage 7 compared to 1355B anthers. The *ACX3* gene has medium-chain-length (C8:0 to C14:0) substrate specificity^50^. These results indicate that the proportion of fatty acid and fatty acid derivatives may change at stage 7 and that de novo fatty acid biosynthesis may be blocked at stage 7 in 1355A anthers, although the fatty acid synthase complex may not be affected at that stage. Lipid stain also validated the results by showing more lipids being deposited in the exine wall at stage 8 (Fig. 3C and G).

Therefore after the tetrad stage, a key period for pollen wall formation^33^,^51^,^52^, abundant sporopollenin synthesis, which is related to the abnormal fatty acid pathway, leads to the 1355A male sterility as shown by failing to form spins on the pollen wall surface, a thicker nexine and lack of intine. Ultimately, defective pollen walls lead to male sterility in 1355A plants.

## Methods

### Plant materials

The recessive genic male sterile (RGMS) two-type line (1355AB) was used as the plant material. The 1355AB line was maintained by full sib-mating (1355A × 1355B); therefore the male sterile line 1355A and the male fertile line 1355B are near-isogenic lines (NILs) differing only in the fertility trait. 1355AB plants were cultivated in the field during the normal cotton planting season and in the greenhouse during the winter in Wuhan, China, using standard farming practices. During anthesis, male sterile plants were identified by staining the pollen grains with I_2_-KI solution and were photographed under a microscope (Leica DM2500, Germany). Bud lengths ranging from 6–7, 7–8, and >24 mm corresponding to stage 7 (tetrad pollen period, TTP), stage 8 (uninucleate pollen, UNP) and stage 12 (mature pollen period, MTP) respectively were collected, and the anthers were sampled for RNA-seq. All of the harvested samples were immediately deep-frozen in liquid nitrogen and stored at −70°C before use.

### Histological analyses

To identify the anther development stage, flower buds were harvested from plants in the field and fixed in FAA [10% formalin, 5% acetic acid, and 50% ethanol (v/v)]. Detailed cross-sections were performed as previously described^53^.

### Lipid staining

Lipid staining was conducted as described by Dun *et al.* (2011)^54^. The anther section of the 1355AB lines during stage 7, stage 8 and stage 9 were deparaffinised by incubating in dimethylbenzene twice for 30 min each and were rehydrated in an ethanol gradient for 5 min. The sections were stained in 1% Sudan Black B in 70% ethanol at 58°C for 45 min. Sections were washed with 70% ethanol to remove excess colour and were observed by light microscopy.

### Illumina sequencing and data processing

Total RNA was isolated from collected anthers using a modified guanidine thiocyanate method^55^. RNA-seq was performed by the Beijing Genomics Institute (Shenzhen, China). mRNA was enriched and broken into short fragments (approximately 200 bp). Using the mRNA fragments as templates, double stranded cDNA was synthesised. After attaching the sequencing adaptors, the library fragments were purified by agarose gel electrophoresis and enriched by PCR amplification, after which the fragments were sequenced (Illumina HiSeq™ 2000). Clean reads were acquired after certain steps of raw sequences data processing.

Clean reads were mapped to the cotton contigs assembly using SOAPaligner/soap2 mismatches; no more than 2 bases were allowed in the alignment. The number of clean reads for each gene was calculated and then normalised to Reads Per Kb per Million reads (RPKM), which associates the read number with the gene expression levels.

### Screening of differentially expressed genes

A rigorous algorithm was used to screen the differentially expressed genes in cotton anther development. A false discovery rate (FDR) ≤ 0.001 and an absolute value of log_2_ Ratio ≥ 1 were used as thresholds to determine the significance of the gene expression difference. A bioinformatics analysis of the gene expression patterns was performed according to Xu *et al*. (2011)^56^.

### Quantitative RT-PCR

Quantitative RT-PCR (qRT-PCR) was used to validate RNA-seq data in identifying genes that were related to 1355A male sterility, according to Xu *et al*. (2011)^56^. Gene-specific primers (Supplementary Table S6) were designed according to the sequences (Supplementary Table S7) with Primer Premier 5 (http://www.premierbiosoft.com/crm/jsp/com/pbi/crm/clientside/ProductList.jsp).

### Electron microscopy

To compare the pollen wall differences between the 1355A and 1355B plants, mature pollen was collected and fixed in 2.5% (v/v) glutaraldehyde. Scanning electron microscopy (SEM) was performed as previously described by Min *et al*. (2013)^57^.

For transmission electron microscopy (TEM), different stages of anthers were dissected, and the middle parts of the anther tissues were immediately prefixed in 2.5% glutaraldehyde (v/v)/0.1 M phosphate buffer (pH 7.2) at 4°C and then vacuum-infiltrated until the samples sank to the bottom of container as described by Zhou *et al*. (2009)^58^. The nexine wall thickness of the microspores was measured using Nano measurer 1.2 software (http://emuch.net/html/201402/7022970.html). The measurements were repeated 10 times for each sample.

## Supplementary Material

Supplementary Information supplementary information

Supplementary Information supplementary table s2

Supplementary Information supplementary table s3

Supplementary Information supplementary table s4

Supplementary Information supplementary table s5

Supplementary Information supplementary table s6

Supplementary Information supplementary table s7

## Figures and Tables

**Table 1:** Summary of tag numbers

	Stage 7		Stage 8		Stage 12		Total
	1355B	1355A	1355B	1355A	1355B	1355A	
Clean reads	7 317 252	7 438 268	7 351 770	7 467 140	7 492 466	7 324 211	44 391 107
Total Mapped Reads	2 851 673	2 873 607	2 755 481	2 704 315	2 635 598	3 128 466	16 949 140
(%) of Total Mapped Reads	38.97%	38.63%	37.48%	36.22%	35.18%	42.71%	38.18%
Unique Match	2 606 158	2 617 802	2 486 204	2 478 338	2 399 000	2 885 941	15 473 443
(%) of Unique Match	35.62%	35.19%	33.82%	33.19%	32.02%	39.40%	34.86%
Total Unmapped Reads	4 465 579	4 564 661	4 596 289	4 762 825	4 856 868	4 195 745	27 441 967
(%) of Total Unmapped Reads	61.03%	61.37%	62.52%	63.78%	64.82%	57.29%	61.82%
