# Supplementary material for: Defective pollen wall contributes to male sterility in the male sterile line 1355A of cotton
Source: Sci Rep. 2015 Jun 5;5:9608. doi: 10.1038/srep09608 (PMC4456728; doi:10.1038/srep09608)
Supplement: Supplementary Information [file srep09608-s1.pdf]

Supplementary information

Defective pollen wall contributes to male sterility in the male sterile line 1355A of cotton

Yuanlong Wu, Ling Min, Zancheng Wu, Li Yang, Longfu Zhu, Xiyan Yang, Daojun Yuan, Xiaoping Guo and Xianlong Zhang*

National Key Laboratory of Crop Genetic Improvement, Huazhong Agricultural University, Wuhan, Hubei 430070, China

Running Title: Defective pollen wall in 1355A

* To whom correspondence should be addressed.
National Key Laboratory of Crop Genetic Improvement, Huazhong Agricultural University, Wuhan, Hubei 430070, China
Tel: +86-27 8728 0510,
E-mails: xlzhang@mail.hzau.edu.cn.

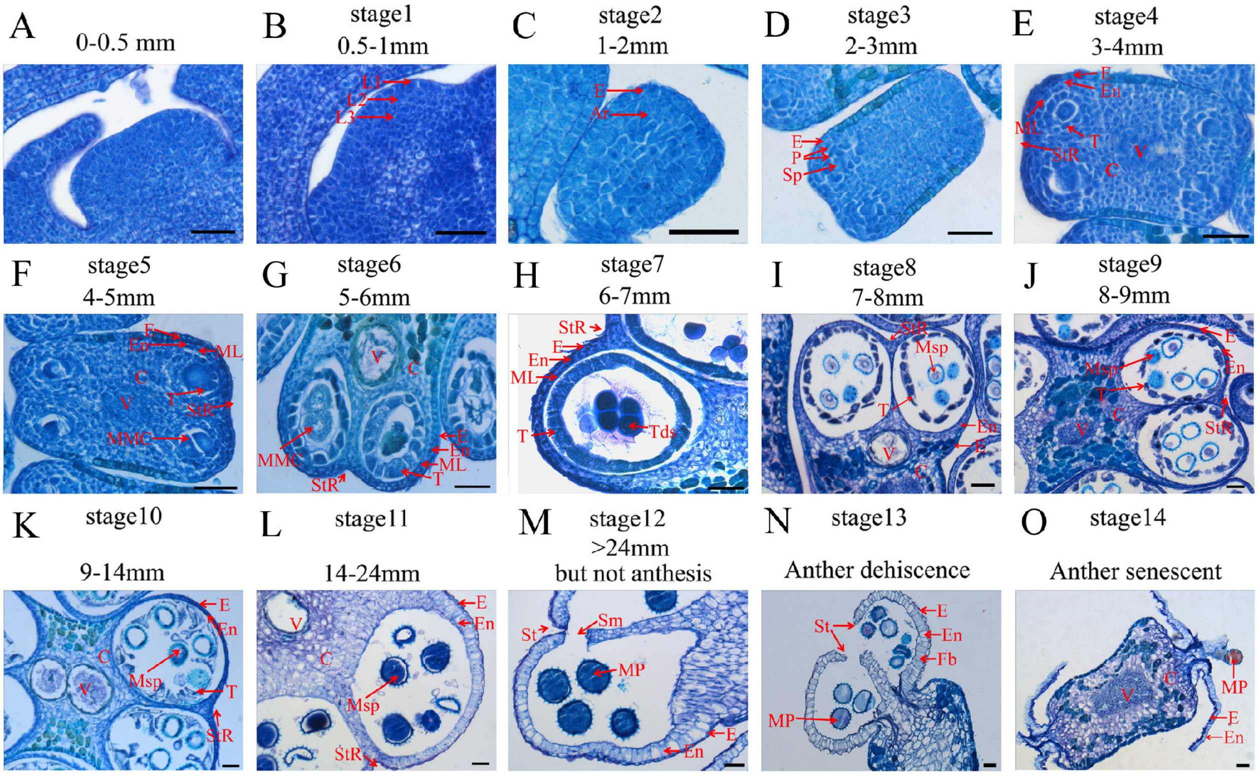

Supplementary Fig. S1. The anther development stages were described in the male fertile line 1355B of *G. hirsutum*. The relationship between the bud lengths and the anther development stages are shown. (A) Buds with lengths of 0-0.5 mm exhibited flower primordia, but anther primordial were not yet evident. (B) Anther primordia emerged at stage 1. (C) The archesporial cells, which form in the L2 layer, were examined at stage 2. (D) At stage 3, anthers exhibited parietal layers and sporogenous cells, which are derived from archesporial cells. (E) At stage 4, the stomium and vascular tissues were generated. (F) At stage 5, the four layers and the microspore mother cells were evident. (G) At stage 6, meiosis occurred in the microspore mother cells. (H) When the meiosis was completed at stage 7, the tetrads were formed. (I) Microspores were released from the tetrad at stage 8. (J) The anther expansion and growth was present at stage 9. (K) The tapetums were degenerated at stage 10. (L) At stage 11, microspore enters mitosis. (M) At stage 12, mature pollen was generated, and the septum below the stomium degenerated. (N) After anthesis, the anthers were dehiscent at stage 13. (O) At stage 14, they were senescent. L1, L2 and L3, the three cell-layers in the stamen primordia; Ar, archesporial cell; P, parietal layer; Sp, sporogenous layer; E, epidermis; En, endothecium; ML, middle layer; T, tapetum; StR, stomium region; C, connective; V, vascular region; Sm, septum; St, stomium; Fb, fibrous bands; MMC, microspore mother cells; Tds, tetrads; Msp, microspores and MP, mature pollen. Bars, 50 μm.

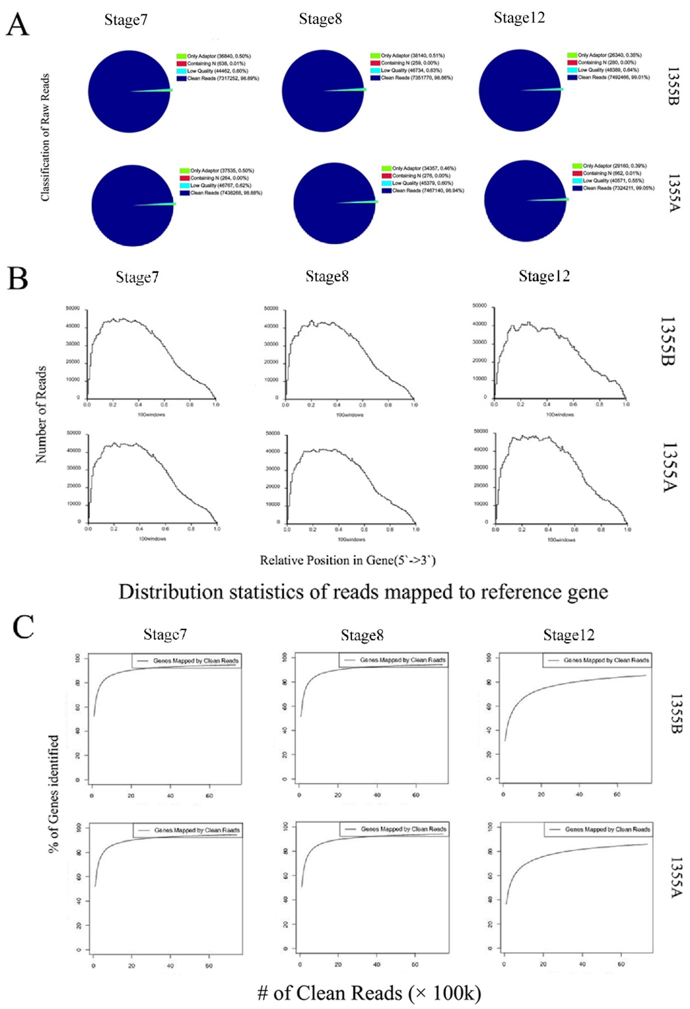

Supplementary Fig. S2. The sequencing assessment.

(A) Sequencing quality evaluation of six different libraries. The raw reads were divided into clean reads, low-quality reads, reads containing N and only adaptor reads. The clean reads were mapped to reference sequences.

(B) The randomness assessment of six different libraries. The distributions of reads locating on the genes were used to evaluate the randomness.

(C) Sequencing saturation analysis of six different libraries. The numbers of identified genes increased with the number of clean reads. When the number of clean reads reached 6-7 million, the six libraries approached saturation.

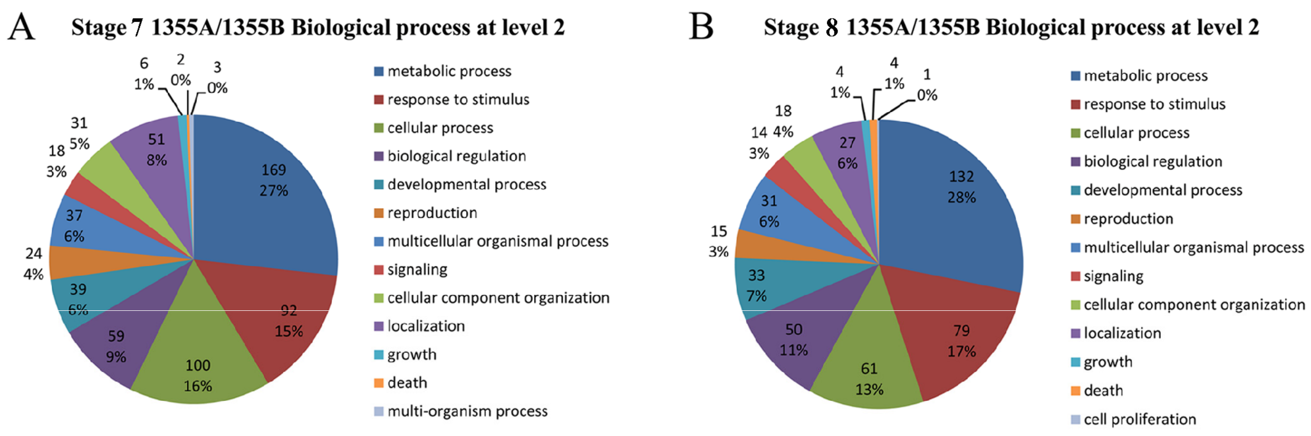

Supplementary Fig. S3. Functional categories of DEGs assigned GO terms at the appropriate level. Compared to those of the 1355B anthers, the DEGs in the 1355A anthers by GO categories during stage 7 (A) and stage 8 (B) in the biological processes were presented at level 2.

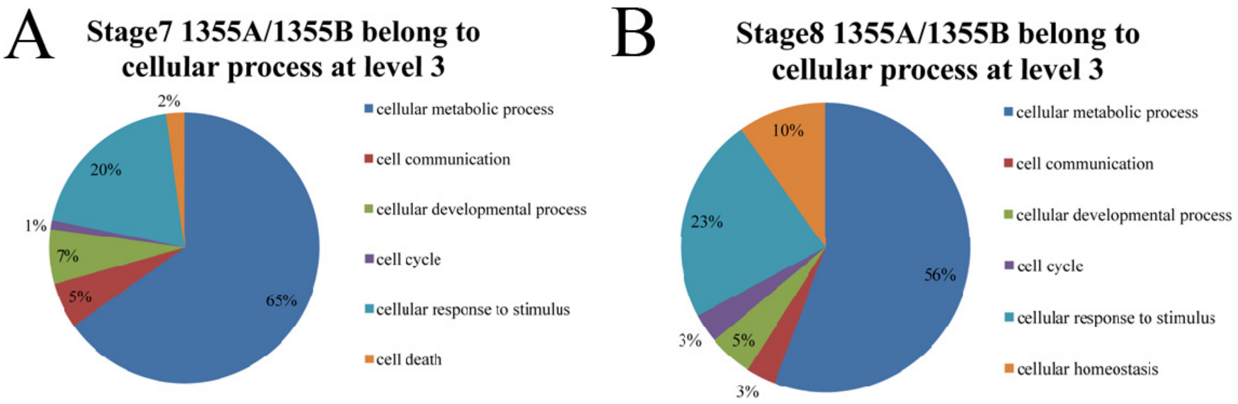

Supplementary Fig. S4. The DEGs in the 1355A anthers of GO categories of the 'cellular process' at level 2 assigned GO terms at level 3. (A) The GO categories of the 'cellular process' genes in biological processes at level 3 at stage 7. (B) The GO categories of the 'cellular process' genes in biological processes at level 3 at stage 8.

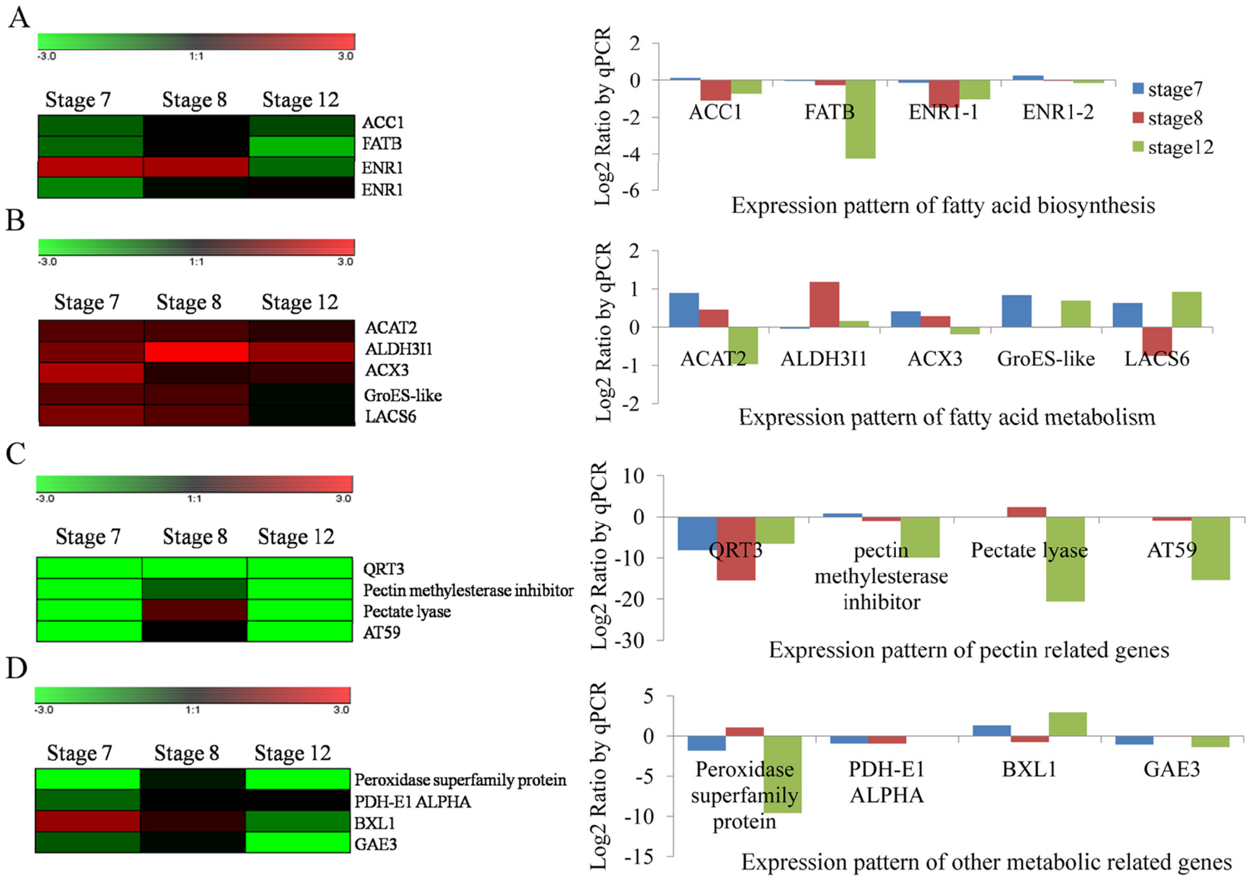

Supplementary Fig. S5. Detailed expression profiles of 17 differently expressed genes in the 1355A plants were compared to those of the 1355B plants. The relative expression level was obtained by RNA-seq after using the equation and logarithmic transformations of RPKM and qRT-PCR for data verification. (A) The fatty acid biosynthesis genes, *ACC1* (acetyl-CoA carboxylase 1, ES840243), *FATB* (fatty acyl-ACP thioesterases B, ES849077), *ENR1-1* (NAD(P)-binding Rossmann-fold superfamily protein, ES806303), and *ENR1-2* (NAD(P)-binding Rossmann-fold superfamily protein, ES818872). (B) The fatty acid metabolic genes, *ACAT2* (acetoacetyl-CoA thiolase 2, ES834402), *ALDH3I1* (aldehyde dehydrogenase 3I1, DW480953), *ACX3* (acyl-CoA oxidase 3, EX164385), GroES-like (GroES-like zinc-binding alcohol dehydrogenase family protein, ES797791) and *LACS6* (long-chain acyl-CoA synthetase 6, ES813531). (C) The pectin related genes, *QRT3* (pectin lyase-like superfamily protein, DW484863), pectin methylesterase inhibitor (EX164972), pectate lyase (EX166210) and *AT59* (Pectate lyase family protein, EX166094). (D) The other metabolic related genes, peroxidase superfamily protein (DT572457), PDH-E1 ALPHA(dehydrogenase E1 alpha, ES824346), *BXL1* (beta-xylosidase 1, ES827195) and *GAE3* (UDP-D-glucuronate 4-epimerase 3, GQ292790).

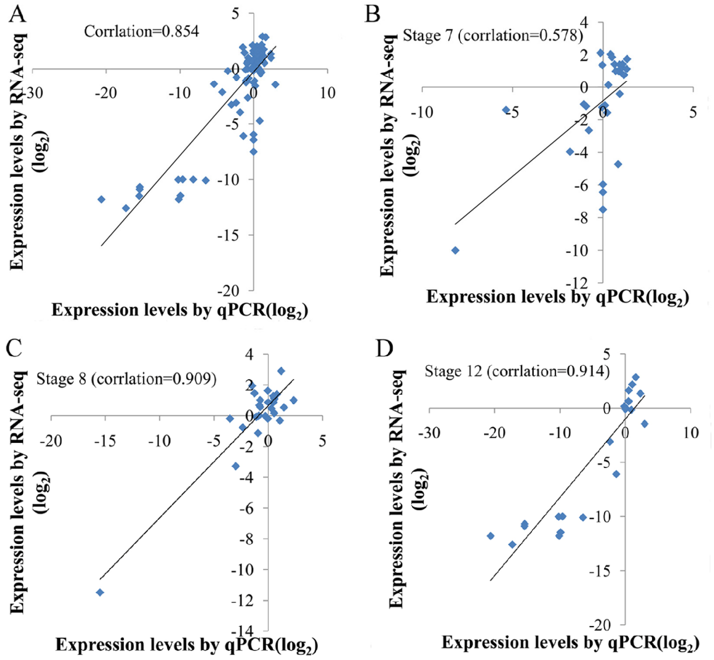

Supplementary Fig. S6. Comparison of gene expression profiles following RNA-Seq and qRT-PCR. A comparison of the expression profiles of 29 selected DEGs in the 1355A by RNA-Seq and qPCR in three stages (A), stage 7 (B), stage 8 (C) and stage 12 (D).

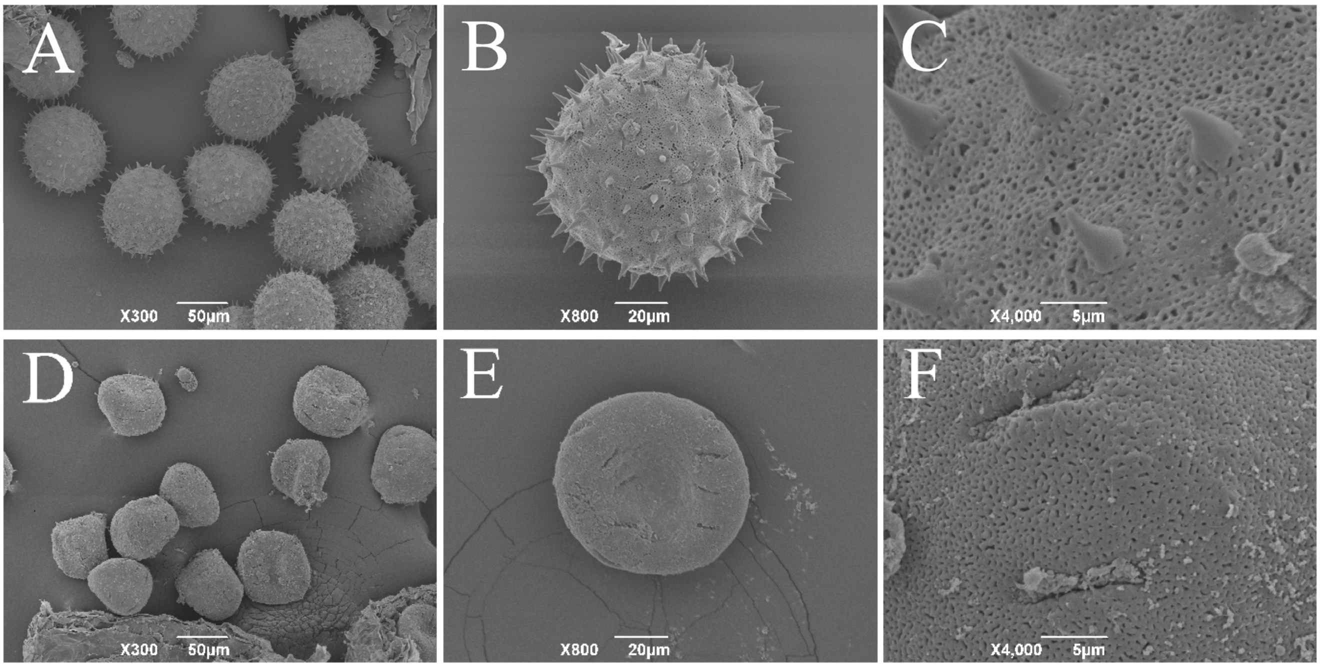

Supplementary Fig. S7. Scanning electron microscopy analysis of pollen grains of the 1355B (A-C) and 1355A (D-F) during stage 12. Compared to the 1355B pollen (A-C), the exine spine was not produced on the surface of 1355A pollen grains (D-F). Bars = 50 µm in (A) and (D), 20 µm in (B) and (E), and 5 µm in (C) and (F).

Supplementary Table S1. Relationship between bud sizes and anther development stages in Arabidopsis and G. hirsutum (Coker 315 and 1355B) based on comparison of cellular events during anther development

**stages**	**Arabidopsis** (Sanders *et al.*, 1999)	**G.*hirsutum*, Coker 315** (Xu *et al.*, 2014)	**Coker 315 floral bud sizes** (Xu *et al.*, 2014)		**G.*hirsutum*, 1355B**	**1355B floral bud length (from the nectary to the top of the bud, mm)**
			**Length (mm)**	**Width (mm)**		
1	Rounded stamen primordials emerge.	/	/	/	Anther primordia emerge.	0.5-1
2	Archesporial cells arise in L2 layer.	/	/	/	The archesporial cells derived from the L2 layer.	1-2
3	The primary parietal and sporogenous layers generate the secondary parietal layers and sporogenous cells, respectively.	The secondary parietal layers and cells are apparent.	4.2-4.4	2.3-2.4	The parietal layers and sporogenous cells are exhibited.	2-3
4	Four-lobed anther pattern with two developing stomium regions generated. Vascular region initiated.	Formation of the epidermis, endothecium, middle layer and tapetum has been initiated. Vascular region appears.	4.4-4.8	3.2-3.5	The stomium and vascular tissues were generated.	3-4
5	Four clearly defined locules established. Microspore mother cells appear.	The microspore mother cells appear.	6.4-6.5	4.1-4.2	The four layers and the microspore mother cells were evident.	4-5
6	Microspore mother cells enter meiosis. Middle layer is crushed and degenerates. Tapetum becomes vacuolated.	Microspore mother cells commence meiosis and the tapetal cells become vacuolated.	7.3-7.6	4.3-4.5	Meiosis occurred in the microspore mother cells.	5-6

7	Meiosis completed. Tetrads of microspores free within each locule. Remnants of middle layer present.	The tapetal cytoplasm is condensed and tetrads appear in the anther locules.	8.6-8.8	5.3-5.6	The tetrads were formed.	6-7
8	Callose wall surrounding tetrads degenerates and individual microspores released.	Microspores are released from the tetrads. The tapetal cell walls have been degraded.	9.7-9.9	6.2-6.4	Microspores were released from the tetrad.	7-8
9	Microspores generate an exine wall and become vacuolated. Septum cells can be distinguished.	**Degeneration of tapetal layer appears to commence.** Microspores are vacuolated.	10.5-10.6	6.8-7.1	Anther expansion and growth.	8-9
10	**Tapetum degeneration initiates.** Less vacuolated microspores.	**The tapetum has been degraded.** Remnants of tapetal cells visible. The microspores are still vacuolated.	12.4-12.8	7.5-7.8	**The tapetums were degenerated.**	9-14
11	Pollen mitotic divisions occur. Tapetum and septum cell degeneration initiates. Stomium differentiation begins.	Early pollen grains appear.	17.1-17.5	10.4-10.8	Microspore mitotic divisions occur.	14-24
12	Anther contains tricellular pollen grains. Anther becomes bilocular after degeneration and breakage of septum below stomium.	/	/	/	The mature pollen was generated, and the septum below the stomium degenerated.	>24 But not anthesis
13	Dehiscence. Breakage along stomium and pollen release.	/	/	/	The anthers were dehiscent.	Early anthesis
14	Senescence of stamen. Shrinkage of cells and anther structure.	/	/	/	Senescence of stamen.	Late anthesis
